# A meta-analysis of COVID-19 vaccines acceptance among black/African American

**DOI:** 10.1016/j.heliyon.2022.e12300

**Published:** 2022-12-10

**Authors:** Rezaul Karim Ripon, Umma Motahara, Adiba Alam, Kifayat Sadmam Ishadi, Md Samun Sarker

**Affiliations:** a Department of Public Health and Informatics, Jahangirnagar University, Savar, Dhaka, Bangladesh; b Antimicrobial Resistance Action Center (ARAC), Bangladesh Livestock Research Institute (BLRI), Savar, Dhaka, Bangladesh

**Keywords:** Meta-analysis, COVID-19 vaccine acceptance, Black/African American

## Abstract

The COVID-19 pandemic had harmed Black/African Americans disproportionately. Mortality and morbidity can reduce by increasing vaccination acceptability and availability. We conducted a meta-analysis of 20 studies that show the prevalence of Black/African Americans who embrace COVID-19 vaccination between 2020 and September, 2022. Investigations conducted before and after the availability of COVID-19 vaccines found the vaccinations effective. The heterogeneity was examined using stratified analyses, the meta-regression approach, and sensitivity analysis in R programming language. This meta-analysis showed that the overall COVID-19 vaccine hesitancy among Black/African Americans is 35% (95% CI: 26%–45%). That means 65% of Black African Americans received vaccines without any hesitancy. According to correlation analysis, there was a negative relationship (r = -0.392, P = 0.021) between the prevalence of vaccine hesitancy and the survey year. Evidence suggests ethnic health disparities in Black/African Americans were for lower socioeconomic status. Some initiatives had to address health disparities, while ethnicity had not consistently been a focus. Only vaccines can prevent COVID-19 like infectious diseases. Policy makers and health educators should concern on vaccine acceptance or hesitancy related programs among Black/African American.

## 1 Introduction

Coronavirus disease 2019 (COVID-19) caused by acute respiratory syndrome coronavirus 2 (SARS-CoV-2), created the pandemic condition. The first COVID-19 patient was diagnosed in the United States (US) in January 2020, and by December 2020, it had become the leading cause of mortality in the US [1]. There were almost 1.9 million confirmed cases worldwide on April 14, 2020, with 601,000 infections and 24,129 deaths in the United States alone [2]. The population must be vaccinated against COVID-19 to achieve immunity, and the vaccination should have a high level of acceptance [3, 4]. Hesitancy toward the COVID-19 vaccine threatens the potential for population protection against COVID-19. Vaccine reluctance encompasses various actions, including vaccine refusal and delayed acceptance [3]. Vaccine hesitancy is linked to sociodemographic characteristics such as age, sex, race/ethnicity, income, and education [5, 6]. Race and ethnicity significantly impact vaccination, including the COVID-19 vaccine [5]. Black African Americans have an excellent standard of vaccine apprehension and a low level of vaccine assurance. The significant amount of vaccine apprehension is problematic in and of itself, but when combined with mounting evidence that COVID-19 disproportionately affects ethnic minorities, it is pretty alarming [6]. Despite having the highest risk of COVID-19 morbidity and mortality in the United States, racial and ethnic minority communities report poorer vaccine confidence and lower COVID-19 vaccination rates [7, 8]. Vaccine hesitancy among Black African Americans is more than double that of whites, and this finding is concerning because COVID-19 hospitalizations and deaths are more common among Black/African Americans [9]. Several discrepancies in health facilities based on race and ethnicity have been observed in the United States, and this gap is worrying in the case of COVID-19 vaccine acceptance [10].

This study will demonstrate a pooled prevalence of acceptability of the COVID-19 vaccine acceptance among Black/African Americans as they have been disproportionately under-vaccinated.

## 2 Methods

### 2.1 Study design

We conducted a meta-analysis to determine the prevalence of COVID-19 vaccination acceptance among black/African Americans. The studies' papers were reviewed using the Preferred Reporting Items for Systematic Reviews and Meta-Analyses (PRISMA) criteria [11]. Analyses based on publicly available data do not require ethics approval or authorization.

### 2.2 Eligibility criteria

Studies that reveal the general proportion of Black/African Americans who embrace COVID-19 vaccination are one of the requirements for admission. COVID-19 vaccines were confirmed to be efficacious in tests conducted before and after they became available. This study looked into COVID-19 vaccines in all forms. The researchers looked for publications that employed cross-sectional, case-control, and cohort designs published in English between 2020 and September, 2022. Case studies, conference papers, proceedings, abstract-only publications, editorial reviews, letters of communication, comments, systematic reviews, and qualitative research were all excluded from consideration. In addition, articles are written in a language other than English excluded. The studies that satisfy the following requirements were included in our analysis [1] Only Black/African Americans with COVID-19 vaccine acceptancy or hesitancy were included in the studies [2], which also studies included the prevalence of vaccine hesitancy or acceptance and [3] clear indication of sample size. According to those studies, only odd ratios/risk ratios/relative risks/mean differences was left out of the analysis (means there is no indication of prevalence on vaccine hesitancy or acceptance prevalence).

### 2.3 Information sources search strategy

For publications published between 2020 and September, 2022, a thorough search of the PubMed, Google Scholar, Scopus, ISI, and Web of Knowledge databases was conducted. The generic free-text search phrases "COVID-19″ AND "vaccine" AND "acceptance" OR "hesitancy" AND "Black/African American" were used in the search. The search was limited to full-text articles written in English. Cross-checking the reference lists of the listed citations yielded additional potentially relevant studies.

### 2.4 Data collection process and data items

**Figure 1:**
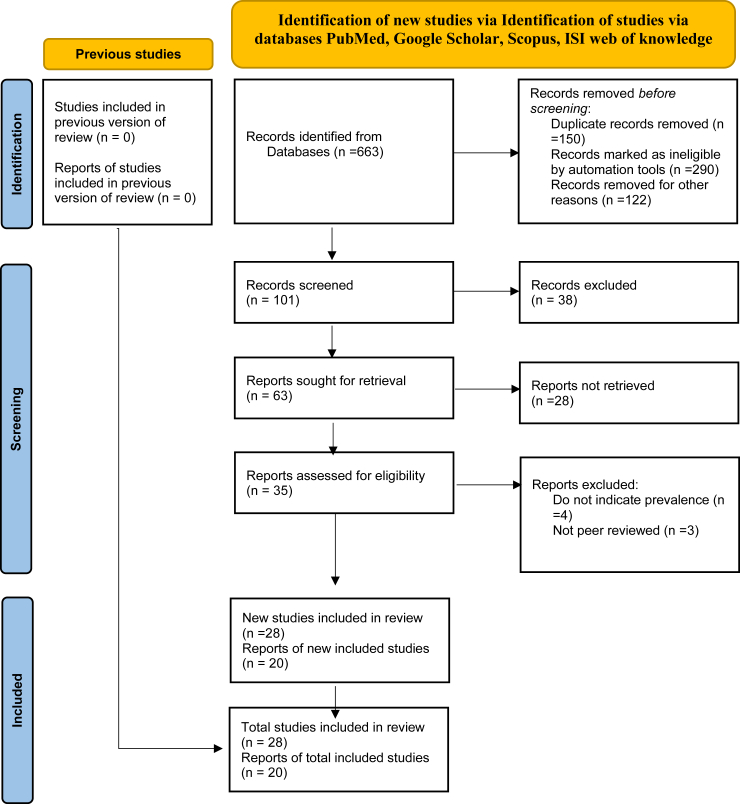
PRISMA flow diagram for this study.

As a result of our search approach, all items were exported to the EndNote program. Duplicated articles were removed from the database. One neutral reviewer (AA) examined the titles and abstracts of the discovered papers. The whole texts of qualifying papers were obtained and appropriately evaluated for eligibility. The records were examined for legitimacy by the second examiner (KSI). In the event of a disagreement between the two reviewers (AA & KSI), the reviewer was then consulted by RKR. Then the articles information was included by two reviewers (SS & UM). And Final checked by one reviewer (RKR). And statistical analysis was performed by RKR and UM. The search strategy was depicted in a PRISMA flow chart, which showed which studies were included and which were excluded and the reasons for exclusion (Figure 1).

### 2.5 Reporting bias assessment

Data quality was assessed according to the Joanna Briggs Institute [12, 13]. Appropriate sample frame, study participants sampled, sample size, description of study subjects and setting, sample size justification, power description, or variance and effect estimates, valid methods for condition identification, a standard and reliable condition measured appropriate statistical analysis, and adequate response rate were among the nine factors used to assess the risk of bias. The phrases "yes," "no," "unclear," and "not available" were used to convey the risk assessment criteria. They received a one-point score, while the others received a zero. The total score was 0–9. The likelihood of bias was considered low when the overall score was greater than 70% (a score of 6 or higher), moderate when it was 50–69%, and high when it was 0–49% [12, 13].

### 2.6 Statistical analysis

After reviewing the entire article for quality, the authors independently extracted data using purpose-built forms that specified the pertinent factors. Disagreements were settled by debating the articles and coming to an agreement. The significant dependent variable was a binary categorization of study results based on whether the article supported the prevalence of vaccine acceptance/hesitancy of Black/African Americans or not. The proportion for the study based on the standardized effect size was the dependent variable in the meta-analysis. The statistical analysis was carried out with the help of the R programming language. The metafor and metareg package in the R programming language was used to determine the pooled prevalence after excluding low methodological quality articles [14, 15, 16]. Metafor uses inverse-variance weights from a random-effects model to pool proportions and offers a weighted sub-group and overall pooled estimate [15, 16]. It entailed a meta-analysis of the prevalence values of each publication, weighted by sample size and allowing for potential heterogeneity across studies in this context [16]. Before random-effects model, Freeman-Tukey transformation was used to produce a proportional meta-analysis [14, 15, 16]. Utilizing a random-effects meta-analysis to account for between-study heterogeneity, prevalence estimates were merged [17, 15, 16]. The statistical heterogeneity was assessed using the chi-square test and on the Q statistic (H & I^2^), which was quantified by the I-square values, under the assumption that I-square values of 25, 50, and 75% were nominally assigned as low, moderate, and high estimates, respectively [17, 14, 15, 16]. To examine potential sources of heterogeneity, the following grouping variables were employed in stratified analyses and meta-regression: sample size, sample type, data collection date, research quality, and sampling technique [17, 15, 16]. In order to establish whether one or more studies had an impact on the outcomes by being excluded one at a time, a sensitivity analysis was also performed [15, 16]. The distribution of the observed studies was visually inspected on a funnel plot in order to determine publication bias [17, 14, 15, 16]. Egger's test was then used to measure the level of bias and then correlation test also conducted [14, 15, 16]. The significance threshold was kept at <0.05.

## 3 Results

PRISMA was used to outline the specific steps of the systematic review and meta-analysis method, and Figure 1 outlined the process of selecting relevant papers. We identified 663 of new studies via databases PubMed, Google Scholar, Scopus, ISI, and Web of Knowledge. Due to duplicate records, ineligible by automation tools and other reasons 628 articles are removed. 7 studies were removed for not indicating prevalence and not peer reviewed. Then we include 28 articles in this study and 8 articles also removed for low methodological quality. Then 20 studies were included in the final analysis. The study included 20 studies with a total of 7384 participants (Table 1). Every study subject is Black/African American (general population and health care workers). The articles were released in the years 2020 through 2022. Eight studies received a score of 9, three got an 8, five got a 7, four got a 6, and eight got a 4, according to the quality assessment standards. The studies are generally deemed to be of acceptable quality, according to the quality scores. Only three of the twenty studies used probability sampling, out of all the nonprobability sample techniques.

**Table 1:** Systematic review information.

Study	Total	Events	Sample	Quality score	Sampling Technique
Matthew C. Sullivan, 2022	109	52	Black	9	Nonprobability
Anna Maria Napoles, 2021	1197	379	Black	9	Nonprobability
Manoj Sharma, 2021	428	208	Black	9	Nonprobability
Olihe Okoro, 2021	183	47	Black	9	Nonprobability
Laura M.Bogart, 2021	101	55	Black	8	Probability
Jana Shaw, 2022	263	87	Health Care worker	9	Nonprobability
Marc F. Stern, 2021	1390	775	Black	7	Nonprobability
Mara Eyllon, 2022	342	82	Black	6	Nonprobability
Jeanette B. Ruiz, 2022	87	35	Black	7	Probability
Julia H.Rogers, 2022	289	104	Black	8	Nonprobability
Ramey Moore, 2021	85	55	Black	9	Nonprobability
Michael Daly, 2022	917	682	Black	7	Probability
Casey L. Daniel, 2022	198	54	Black	7	Nonprobability
Yingjie weng, 2021	116	69	Black	8	Nonprobability
Kiefer et al., 2022	456	80	Black	7	Nonprobability
Carroll et al., 2022	325	41	Black	9	Nonprobability
M. Purnell et al., 2022	210	21	Black	8	Nonprobability
Cepeda et al., 2022	346	64	Black	7	Nonprobability
Johnson et al., 2021	248	46	Black	9	Nonprobability
Jones et al., 2021	94	26	Black	6	Nonprobability
Don E. Willis, 2021	1475	39	Black	4	Nonprobability
Amyn A. Malik, 2020	672	40	Black	4	Nonprobability
Don E. Willis, 2021	1205	50	Black	4	Nonprobability
Germann et al., 2022	456	28	Black	4	Nonprobability
Ruiz & Bell 2021	804	52	Black	4	Nonprobability
Bass et al., 2021	501	38	Black	4	Nonprobability
Benis, Seidmann 2021	1644	17	Black	4	Nonprobability
Fontenot et al., 2021	772	10	Black	4	Nonprobability

**Figure 2:**
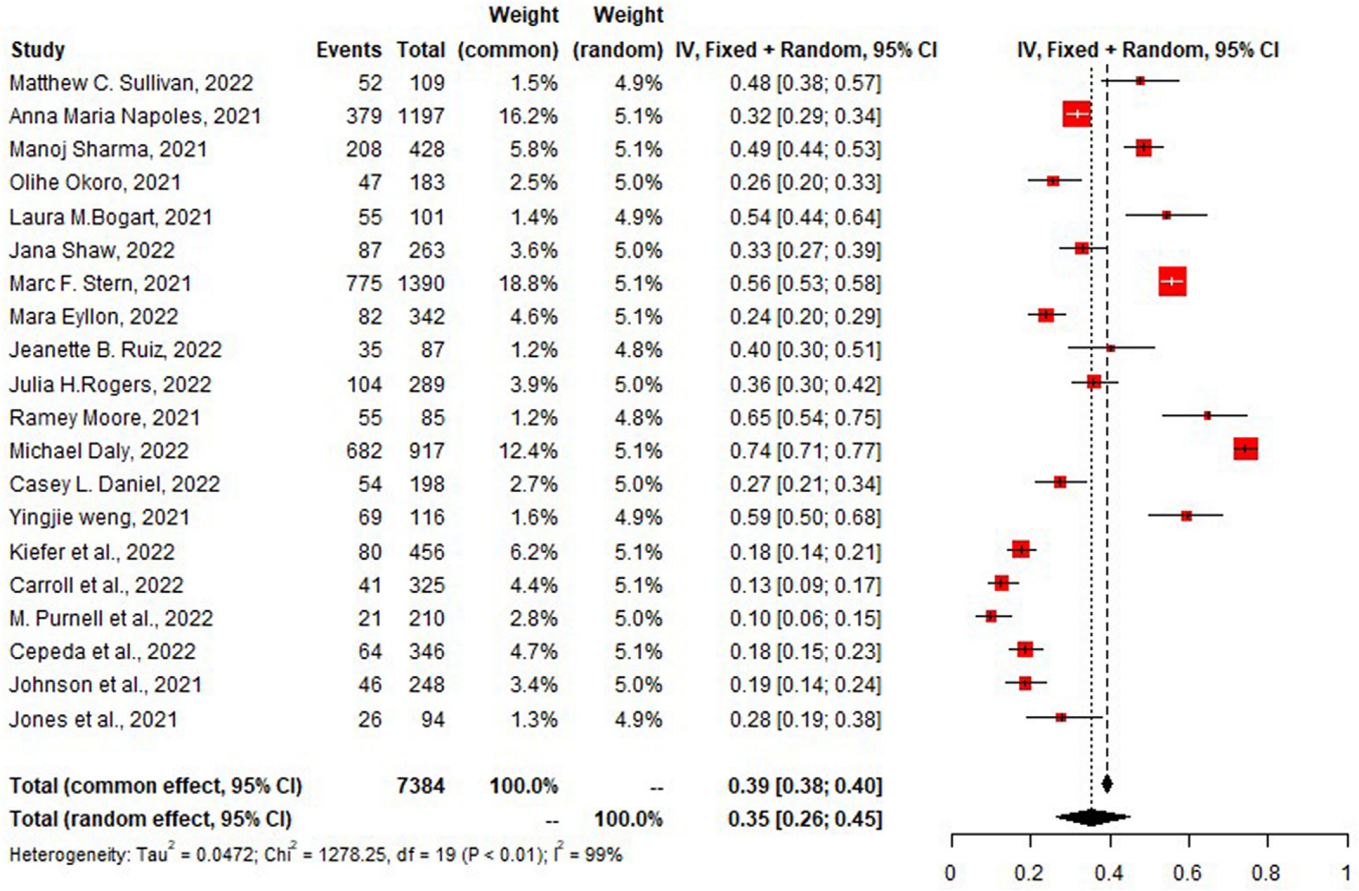
A forest plot of meta-analysis among Black/African American.

**Figure 3:**
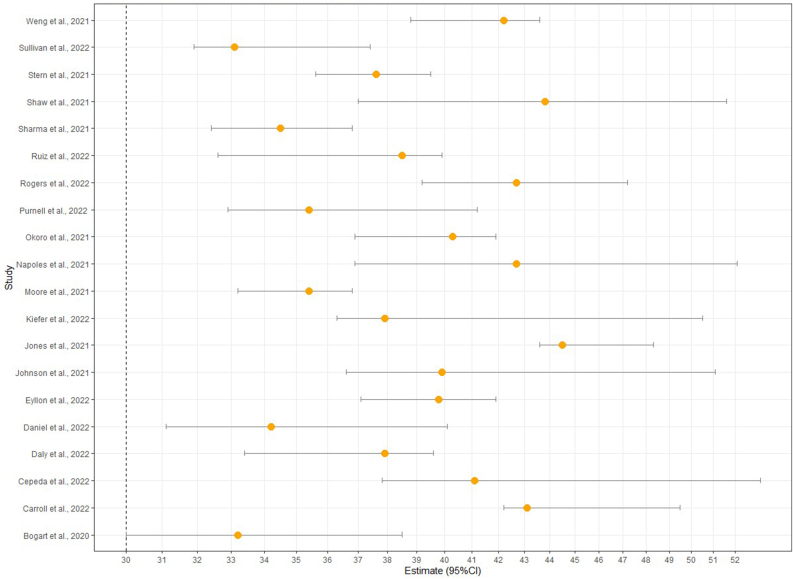
Sensitivity analysis for individual studies.

**Figure 4:**
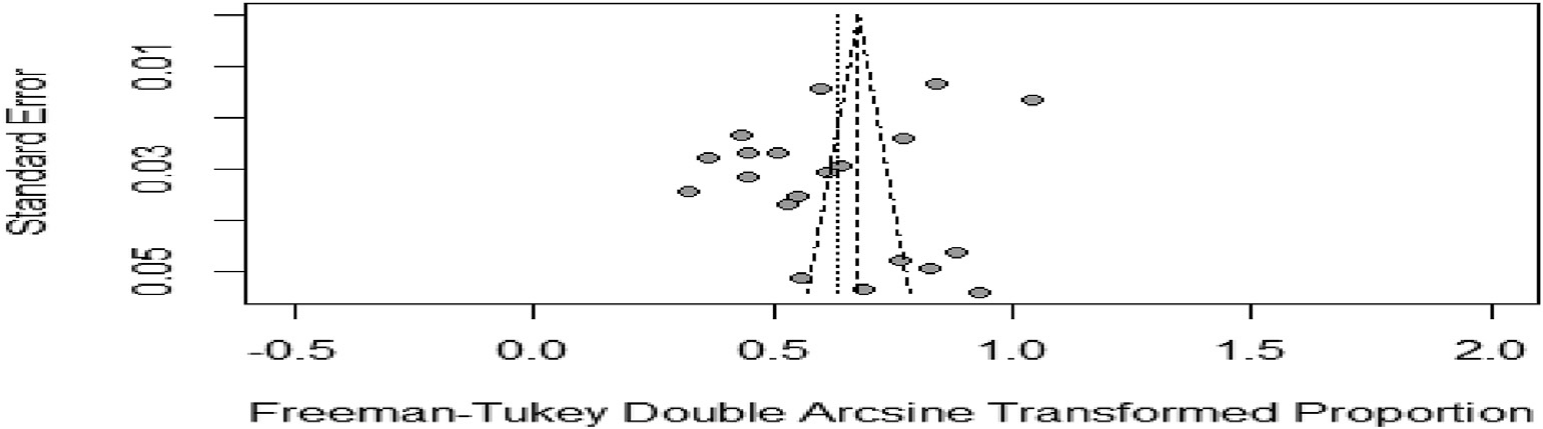
A funnel plot of bias.

We included 20 studies in this meta-analysis. This analysis of show that the overall COVID-19 vaccine hesitancy among Black/African American was 35% (2962/7384 individuals, 95% CI: 26%–45%), with significant evidence of between study heterogeneity (chi square = 1278.25, P < 0.001, I^2^ = 99%) (Figure 2). That means 65% of Black African Americans received COVID-19 vaccines without any hesitancy. Sensitivity analysis was carried out by calculating pooled vaccination hesitancy prevalence once more when any single study was eliminated in order to verify the stability and liability of the meta-analysis. The related pooled vaccine hesitancy prevalence was shown range from 33.2% (30.0%–38.5%) to 43.8% (37.0%–51.6%) without significantly changing (Figure 3). The statistically identical findings indicated that no single study had an impact on the stability of the overall estimate of the prevalence of vaccine reluctance in this meta-analysis. Even though the funnel plot's visual inspection exhibited asymmetry (Figure 4), Egger's test demonstrated substantial value (p = .654), and there was no chance of publication bias.

There was considerable variation between studies. In order to investigate potential sources of heterogeneity, meta-regression (univariate) was used. The meta-regression method was used to examine the sample size, sample type, study period, study quality, and sampling strategy as potential sources of heterogeneity. Table 2 contains the meta-finding on regressions. None of the aforementioned factors were found to be substantially correlated with the identified heterogeneity through the regression model, with the exception of research study time (P = 0.004). Therefore, we conducted additional research to examine the relationship between vaccine reluctance prevalence and probable causes. The prevalence of vaccine reluctance was found to be negative correlated with the research study period (r = -0.392, P = 0.021) (Table 3). That mean vaccine hesitancy may reduce after a time period.

**Table 2:** Result of meta regression.

Covariate	Beta (β) (95%CI)	P value	R^2^ (%)
Sample size	0.0001 (-0.0001–0.0004)	0.3539	12.02%
Sample Technique	0.0001 (-0.0001–0.0005)	0.3453	Nill
Study Quality	0.0436 (-0.2328–0.3200)	0.7571	Nill
Study period	0.1433 (0.0406–-0.459)	0.0039	34.14%
Sample Type	0.0429 (-0.2236–0.3094)	0.7523	1.73%%

**Table 3:** The correlation between the vaccine hesitancy prevalence and potential sources.

Covariate	Sample size	Sample Type	Study period	Quality score	Sampling Technique
p	.513	.123	0.021	.093	.073
r	.632	.432	0.392	.534	.645

## 4 Discussions

Identifying and reducing vaccination hesitancy in COVID-19 is crucial for public health and health disparities, and the pandemic highlights these issues [18, 19, 20, 21, 22, 23, 24, 25, 26, 27, 28, 29, 30]. Low-risk insight is associated with vaccine acceptance [31]. Vaccine receptivity is linked to the trepidation of infection and broad distrust of immunizations [6]. Initiatives must address the history of racism that has eroded credibility in health and medical science organizations among traditionally marginalized communities shattered faith in COVID-19 vaccines and fostered grossly unequal healthcare coverage [32, 33]. COVID-19 vaccination apprehension was higher among racial and ethnic minorities, and Black individuals in the United States were more unlikely than White respondents to take the vaccine [33]. A study reported in the state of Arkansas, where the incidence of COVID-19 vaccination reluctance was most significant among Black/African Americans (50 %), and the odds of COVID-19 vaccine unwillingness were 2.42 times higher for Black/African American respondents assimilated to white respondents (p-0.001) [6]. Another study showed that even though Blacks are disproportionately affected by the virus, they are 40% less interested in seeking a vaccine. Once immunization begins, this could worsen current health disparities [34]. Although Black/African American women are less vaccinated than other races [35]. One study on Black found that women are 72% more likely to avoid vaccination, implying that measures to increase vaccination attitudes in this group should be a significant focus [36]. Immunization endorsement and acceptability by social referents and health practitioners were linked to women's intention to take the vaccine in seven studies [37]. In another study, 11% of women with negative aspirations expressed embarrassment regarding obtaining a vaccine connected with a sexually transmitted disease [37]. The vulnerable group of Black/African American (Children, pregnant women etc) are vaccinated on few days later [38]. The most widespread refusal causes were a lack of evidence about COVID-19 vaccine safety in pregnant populations and the likelihood of injury to the fetus. Pregnant women had greater acceptance of COVID-19 vaccination in the first trimester than in the second and third trimesters [39].

This proportion meta-analysis shows that the overall COVID-19 vaccine hesitancy among Black African Americans was 35% (95% CI: 26%–45%). More than half of Black African Americans received vaccines without hesitancy (Figure 2). A study in 2016 on Black indicates social determinants are improving [40]. Social determinants are linked to vaccine acceptance [6]. Whereas another Global Rapid Systematic Meta-Analysis indicated that the overall COVID-19 vaccination acceptance rate in the African region was 42.46% (95% CI: 23.10–61.82) among the general population, while it was 27.70% among healthcare workers (95% CI: 24.16–31.24) and Cameroon had the poorest approval rate at 15.40% (95% CI: 13.99–16.81) [39]. In contrast, the general acceptability rate of the COVID-19 vaccination was determined to be in European and American regions was 67.04% (95% CI: 60.58–73.50) and 64.77% (95% CI: 57.23–72.32). Regarding the precise explanations for apprehension, the most common concerns of people of all races and ethnicities were long-term adverse effects (50–57 %) and unpleasant reactions (45–54 %). Furthermore, Black and Hispanic people were more likely than White people (37–42%) to say they did not know enough regards the vaccine (45–51%) [33].

Another finding of our study was to negative correlation of vaccine hesitancy and study period. Vaccine hesitancy is reducing among black African American after a time period but it is higher than others races [40]. Our study also indicates there was higher heterogeneity for time period among Black/African American vaccine hesitancy. Vaccine hesitancy can be reduced in a defined time period. But for that, the Government should take some initiative early to reduce the severity of diseases. By that Black people who had hesitancy to get vaccines can trust in vaccines, delivery methods, and health care [41]. Nevertheless, there are cogent reasons why Black people in the United States would be skeptical of a COVID-19 vaccination, and these causes are racist. Subconscious biases within healthcare systems and healthcare professionals have been linked to decreased quality of care and even worse medical outcomes among Black people in the United States [42]. To delay the pandemic, tailored health communication techniques that successfully meet the subpopulations most prone to oppose COVID-19 vaccination and work to alleviate the vaccine-core hesitant fears using scientific evidence would be required [34].

Vaccine hesitancy poses dangers to both the individual and his or her community, since exposure to a contagious disease places the person at risk, and individuals are far more likely to spread the disease to others if they do not get vaccinated [43]. Even though inventing a vaccine which is secure, effective, and affordable is extremely hard, vaccine hesitancy presents a distinct obstacle for academics, professionals in the medical field, as well as members of the public administration and local communities [43]. Many experts have recommended strategies to address vaccine hesitancy at the population level, which include accountability in decision-making regarding vaccination policies, educating the general public and health care professionals about the sophisticated procedure that ultimately resulted in the approval of new vaccines, and diversified post-marketing surveillance of vaccine-related events [44]. Naturally, those who want to undermine public trust in vaccinations will always find methods to disseminate misleading information. However, if given the right guarantees, a substantially larger section of the population could be inclined to get vaccinations, and when their worries are disregarded, they frequently turn to others for support [45].

The main strength of this articles was the pooled prevalence of COVID-19 vaccine hesitancy/acceptance among Black/African American. We solely refer to the Black/African American subgroup. There was the only one study specifically mentions Black/African Americans with social determinants. Because of this, it was not possible to include any other subgroup (based on demographic information) in this study. We looked through numerous databases and websites to discover all relevant and gray publications in order to eliminate database bias but there may be massing some databases or websites. The limitations mentioned above, as well as publication bias and heterogeneity for some of the pooled results, must all be taken into account when interpreting the results.

## 5 Conclusions

Establishing intervention and preventative techniques that focus on changing people's perceptions of vaccination's benefits while simultaneously removing existing barriers is crucial. Governments and policy makers should ensure that people have enough information, a positive attitude, and favourable perceptions about COVID-19 vaccines. Although the vaccine hesitancy less than 50%, the healthcare institutions should implement urgent health promotion programs and disseminate more reliable data on vaccine production process, efficacy and effectiveness. With these activities, vaccine hesitancy may more reduce. In future to increase acceptance, vaccine have available may without any cost.

## Declarations

### Author contribution statement

Rezaul karim Ripon: Conceived and designed the experiments; Performed the experiments; Analyzed and interpreted the data; Contributed reagents, materials, analysis tools or data; Wrote the paper.

Umma Motahara: Performed the experiments; Analyzed and interpreted the data; Contributed reagents, materials, analysis tools or data; Wrote the paper.

Kifayat Sadmam Ishadi, Adiba Alam: Contributed reagents, materials, analysis tools or data; Wrote the paper.

Md Samun Sarker: Contributed reagents, materials, analysis tools or data.

### Funding statement

This research did not receive any specific grant from funding agencies in the public, commercial, or not-for-profit sectors.

### Data availability statement

Data will be made available on request.

### Declaration of interest's statement

The authors declare no conflict of interest.

### Additional information

No additional information is available for this paper.
